# Fully Electrical Post-Fabrication Trimming of Resistive Sensors

**DOI:** 10.3390/s22030767

**Published:** 2022-01-20

**Authors:** Ibrahim Shankhour, Jad Mohdad, Frédérick Mailly, Pascal Nouet

**Affiliations:** LIRMM, University of Montpellier, CNRS, 34000 Montpellier, France; ibrahim.shankhour@lirmm.fr (I.S.); jadmodad@gmail.com (J.M.); frederick.mailly@umontpellier.fr (F.M.)

**Keywords:** calibration, trimming, MEMS, offset, PSRR, resistive sensor, thermal drift of offset

## Abstract

A compact and efficient IC architecture is presented as an alternative to laser-trimmed precision thin-film resistors or look-up tables. The objective is to keep the device, such as a four-terminal Wheatstone bridge, but to compensate for post-manufacturing offset and to avoid the so-induced degradation of performances in terms of full-scale, non-linearity, power supply noise rejection and scale factor. Expected advantages are a reduced cost due to the electrical-only implementation and a possible on-field calibration of high-end sensors. Application of the proposed solution is illustrated on an example to demonstrate improvement factors on offset and sensitivity accuracy of 32 and 10, respectively. Additionally, the power supply rejection ratio is improved by 30 dB. The experimental results finally demonstrate both efficiency and versatility of the proposed solution thanks to a first silicon prototype, fabricated in a 0.35 μm Technology from AMS, connected to an off-the-shelf pressure sensor.

## 1. Introduction

Nowadays, MEMS-based or micromachined resistive sensors have become widely used in many fields of application such as automotive, medical, defense, space, etc. While their consumer counterparts may afford being affected by process- and mismatch-induced scatterings, high-end resistive sensors require precise control of offset, temperature drift and scale factor after post-manufacturing calibration. Classically, a resistive sensor delivers an electrical signal at the output of a Wheatstone bridge. This simple and cheap conditioner is efficient in the absence of mismatch between resistances composing the bridge. However, microfabrication and MEMS-based manufacturing induce an offset, which directly impacts Power Supply Rejection Ratio (PSRR) and thus the minimum detectable signal [1]. Offset also affects the measurement range and non-linearity due to 2nd-order effects. Additionally, scale factor uncertainties also require post-manufacturing calibration, and lastly, temperature may affect offset at the output of the Wheatstone bridge. To cancel-out these imperfections and to improve the grade of a sensor, two state-of-the-art solutions are commonly employed.

The first solution consists of using a set of laser-trimmed resistors connected in series and in parallel with Wheatstone bridge resistors [2,3,4,5]. A costly and individual procedure is then required to measure offset and scale factor of each sensor before calculating the proper value of trimming resistors and a procedure involving numerous cycles of offset and sensitivity measurement followed by laser-based adjustment of trimming resistors is required. Additionally, particular attention must be paid to the thermal drift of offset (TDO) that may appear due to the sensor itself or to the different temperature coefficients of resistance (TCR) between the sensors and trimming resistors. Finally, each product comes out as a batch-fabricated die, i.e., the sensor, and a customized laser trimmed die with compensation resistances in the same package. The main advantage of this solution is that the so-obtained sensor behaves similarly to an uncompensated Wheatstone bridge.

The second solution consists of using a more complex conditioner to convert the physical magnitude into a frequency, a PWM or a digital signal [6,7,8]. Once the signal is in either time or digital domains, calibration coefficients may be stored in look-up tables to allow further compensation of offset, scale factor error or thermal drift of offset. This solution leads to a digital sensor system and, even if efficient, the sensor is no more consistent with an existing equipment that requires a sensor with an analog output such as the differential voltage of a Wheatstone bridge.

This paper proposes a smart and cost-effective alternative to laser trimming based on digital potentiometers [9,10,11]. In [11], a hardware solution with discrete components was presented. This leads to a bulky implementation that is not compliant with most of the standard sensors in which compensation must be included in the same package as the sensor. In a previous paper [12], the principle of a tinny Integrated Circuit (IC) that may digitally control offset, scale factor and temperature drift has been proposed to be integrated in the same package than the sensor as an alternative to a laser-trimmed resistor die. The challenge was then to comply with integration constraints such as resistance values, which must be limited, power-consumption, programmability and non-volatility without any change in the external connectivity of the Wheatstone bridge. 

In this paper, we extend our preliminary proposal [12] with in-depth performance analysis of the solution that leads to the fabrication of a silicon prototype and to a proof of concept with experimental validation. Calibration procedure and non-volatility are not addressed in this paper, as digital tuning words may be easily stored inside the IC using fuses, anti-fuses or non-volatile memories [13,14,15]. It is worth noting that the so-obtained sensor, once connected to the IC, behaves as a bare Wheatstone bridge, thus allowing the replacement of fully passive sensors by their smart counterpart. 

The paper is organized as follows. First, intrinsic performance limitations introduced by mismatches due to manufacturing are presented for a bare resistive sensor in a Wheatstone bridge conditioner. Section 3 presents the principle of the proposed architecture as an efficient alternative to laser trimming for offset compensation. The effect of temperature on the residual offset is then illustrated, and a solution to cancel temperature-induced drift of offset is presented. An embedded infrastructure for fine-offset compensation at each power-up and scale factor adjustment are then presented in Section 4 and Section 5, respectively. Proof of concept with respect to manufacturing-induced processes and mismatch scatterings is demonstrated using Monte Carlo simulations. Finally, experimental results on a first silicon prototype, fabricated in a 0.35 μm Technology from AMS, connected to an off-the-shelf pressure sensor are given in Section 6.

## 2. Wheatstone Bridge Limitations

**Figure 1:**
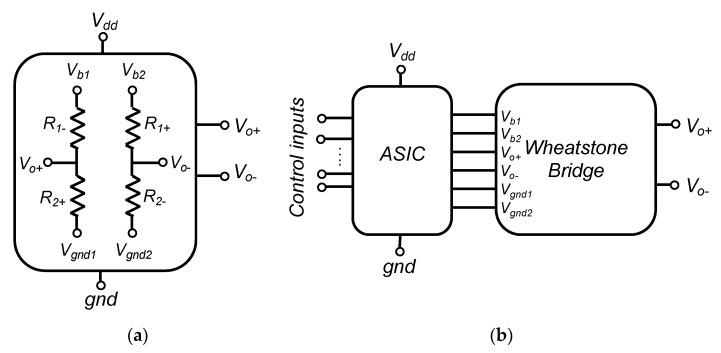
(**a**) Wheatstone bridge arrangement of a resistive sensor, (**b**) Wheatstone bridge connection to the « soft » trimming ASIC.

The Wheatstone bridge (WB) is the most common conditioner for resistive sensors. Sensor resistors are typically organized as depicted in Figure 1a, where terminals *V_b_*_1_ and *V_b_*_2_ are connected to the power supply voltage *V_dd_*, whereas *V_gnd_*_1_ and *V_gnd_*_2_ are connected to the ground (*gnd*). Depending on the sensor architecture, one to four of the WB resistances are sensitive to the physical input, while others are reference resistors with fixed values. Some non-exhaustive examples are:

- Sensors with a single sensitive element: *R*_1−_ = *R*_2−_ = *R*_2+_ = *R*_0_ and *R*_1+_ = *R*_0_ + Δ*R*;
- Differential sensors: *R*_1−_ = *R*_2+_ = *R*_0_, *R*_1+_ = *R*_0_ + Δ*R* and *R*_2−_ = *R*_0_ − Δ*R*;
- Full bridge sensors: *R*_2+_ = *R*_1*+*_ = *R*_0_ + Δ*R* and *R*_1−_ = *R*_2−_ = *R*_0_ − Δ*R*.

where *R*_0_ and Δ*R* are, respectively, the nominal value of resistance and the variation of sensitive resistances induced by the physical magnitude to be measured.

The small-signal differential output voltage of the bridge is then given by:

(1) $$V_{out}=V_{o+}-V_{o-}=\alpha V_{dd}\frac{\Delta R}{4R_{0}}$$

where *α* is the number of sensitive resistors in the bridge, i.e., 1, 2 or 4 in most of the cases. From this equation, the advantage of a WB arrangement is obvious: the differential output voltage is equal to zero when the physical signal is null, thus insuring a good power supply rejection ratio (PSRR) and a high resolution only limited by the signal-to-noise ratio of the bridge. Additionally, if the four resistances of the WB exhibit the same temperature behavior, temperature effects are cancelled.

However, process mismatches, during sensor manufacturing, lead to an unbalanced bridge in the absence of input signal. Post-fabrication calibration is then often required for high-end sensors to cancel those random effects on each sensor. This is classically implemented using an additional die with resistances that may be laser trimmed and placed in series or in parallel with WB resistors. Our proposal, illustrated in Figure 1b, consists in replacing this passive die by an active ASIC that smartly implements all the required adjustments.

**Figure 2:**
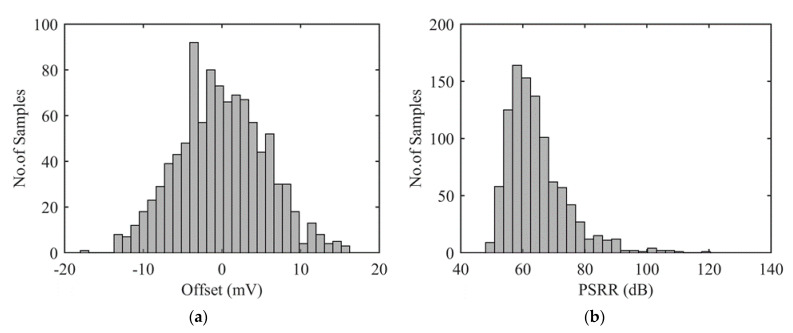
Impact of resistance mismatches on two performances of a Wheatstone bridge: (**a**) Offset in mV and, (**b**) power supply rejection ratio measured at 1 kHz in dB.

Let us first put in evidence limitations of a bare WB. Figure 2a illustrates output voltage offset for a Wheatstone bridge with 4 identical resistance *R*_0_. Monte Carlo (MC) simulations have been performed using Cadence-Virtuoso© design environment taking into account typical mismatches for a standard microfabrication process, i.e., a gaussian distribution of mismatches with σ = 0.23% affecting WB resistors independently. It is worth noting that process variations that affect all four resistances identically do not impact WB offset (Equation (1)). Under a 5 V power supply voltage, a worst-case offset of less than 18 mV is observed. The so-obtained maximum input-referred offset corresponds to 0.36% of signal for a full bridge. Even if this offset lies in the specification of the considered application, it also induces a strong degradation of PSRR as illustrated in Figure 2b. In the worst case, offset is close to 18 mV and PSRR is as low as 48 dB, thus limiting minimum detectable signal in the WB. Obviously, it is also verified that as large is offset, as low PSRR is.

As a conclusion for this preliminary study, small signal detection in a WB is limited by offset and PSRR. State of the art solutions have been presented in the introduction and our proposal consists in adding a compact IC inside the sensor package to allow a fully electrical calibration procedure. This will allow the so-obtained smart-sensor, after in-factory calibration, to behave like a classical passive Wheatstone Bridge in terms of output signals and external connections without offset-induced limitations.

## 3. Offset and Thermal Drift of Offset Compensation

The proposed solution consists in connecting sensor resistors to a calibration ASIC as depicted in Figure 1b. As a result, CMOS die and sensor are packaged as a single device with four external I/O (namely, *V_o+_*, *V_o−_*, *V_dd_* and *gnd*), while few control inputs are used only during post-fabrication calibration. 

### 3.1. Basic Principle for Offset Compensation

**Figure 3:**
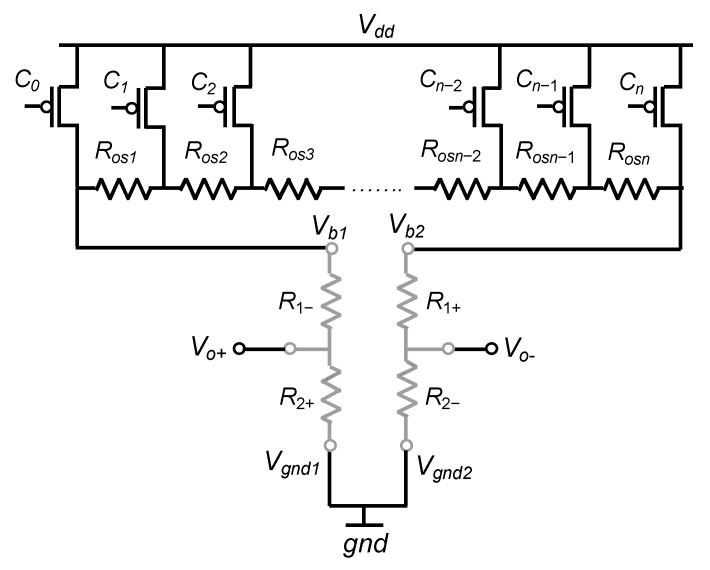
Basic architecture for offset compensation.

The straightforward idea, to compensate for process-induced offset, is to connect a digital potentiometer between *V_dd_* and both terminals of the Wheatstone bridge as depicted in Figure 3. 

There are different ways to control switches (*C*_0_ to *C_n_*) to compensate for post-fabrication offset. The calibration procedure presented hereafter reduces the impact of the on-state resistance of MOS transistors and is easy to implement:

- Initially, all transistors are in the on-state; differential output *V_out_* is measured in absence of signal.
- If *V_out_* is positive (respectively negative), a resistance must be serially added to *R*_1−_ (resp. *R*_1+_). The switch controlled by *C*_0_ (resp. *C_n_*) is opened to add *R_os_*_1_ (resp. *R_osn_*) in the path to *V_dd_*.
- Switches are successively opened from left to right (resp. right to left) until a change of *V_out_* sign is obtained. The configuration code that gives the best offset can then be chosen between the first before the offset sign changes and the first after the sign changes. The maximum offset is then reduced by a factor 2*n*, where *n* is the number of compensation resistors *R_os_*.

The so-obtained algorithm converges very quickly and linearly with *n*. Convergence time has an upper limit equals to *n* periods of the calibration controller clock which maximal frequency must be fixed accordingly to the time constant set by output resistance of the WB and its load capacitance. Few MHz are then acceptable for most sensors, but in an extreme case, e.g., output resistance of 100 kΩ and load capacitance of 100 pF, sampling frequency should be reduced down to 16 kHz. In this later case, and if *n* is such that convergence time would impact calibration cost significantly, dichotomy can be used to reduce this impact.

As an example, let us try to compensate the offset observed in Figure 2. If the maximum offset to be compensated is equivalent to 0.36% of *R*_0_ in a full bridge, a resistance of about 1.5% (≈4 × 0.36%) must be added in one branch or the other depending on the offset sign. If this resistance is discretized in 7 elements (*n* = 7), then each elementary resistance *R_o_*_s_ must be equal to about 0.21% of *R*_0_. 

Monte Carlo simulations are then performed to verify efficiency and results, reported in Table 1, show a reduction of maximum offset by a factor of about 12 (≈2*n*) and a reduction of standard deviation from 5.7 mV down to 766 µV. PSRR is also improved to increase, in the worst case, from 48 dB up to 70.5 dB. It is worth noting that PSRR remains stable over a large band of frequencies. Obviously, increasing the number of elementary resistors may increase performance in terms of residual offset and PSRR with a linear impact on the calibration time.

**Figure 4:**
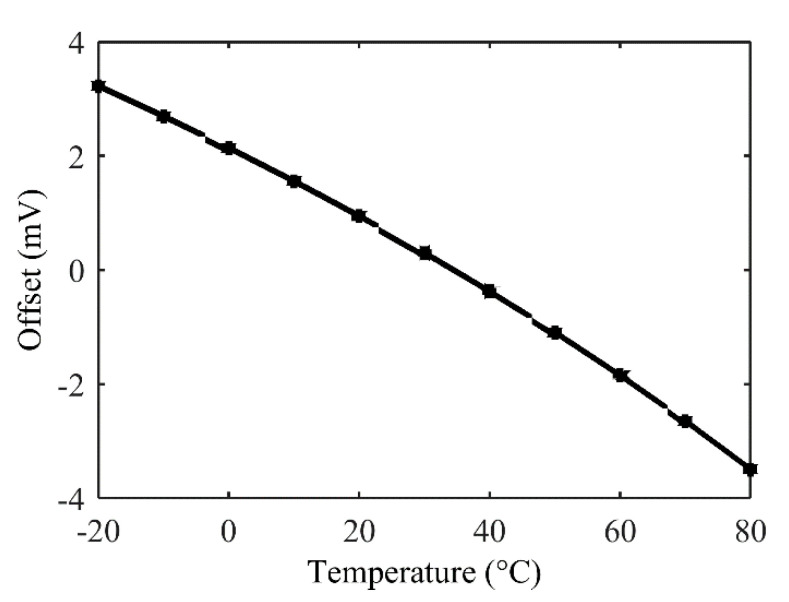
Thermal drift of offset after offset compensation with architecture of Figure 3.

However, this offset compensation architecture induces a thermal drift of offset. Indeed, sensor’s resistances, *R*_0_, and compensation resistances, *R_os_*, will have different TCR and, as illustrated in a typical example (Figure 4), a TDO of about 6 mV is observed over a moderate range of temperature from −20 °C up to 80 °C after compensation of offset. This drift, which is null in a bare WB, can be as high as 6.5 mV over a set of 1000 Monte Carlo simulations of an offset-compensated bridge. 

### 3.2. Thermally Stable Offset Compensation Architecture

To reduce thermal drift, one obvious possibility consists in implementing, inside the IC, resistors with same TCR as the sensor’s resistor one. This solution is not efficient in practice as two different fabrication processes are used for the sensor, on one side, and for the IC, on the other side. 

**Figure 5:**
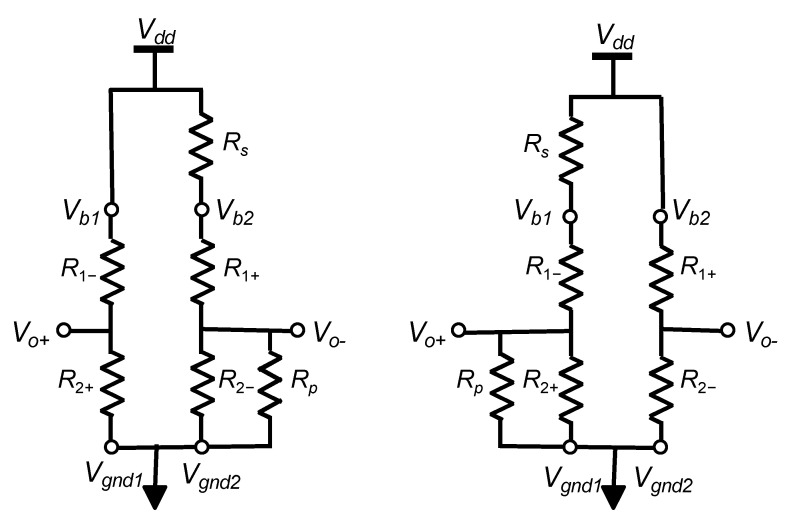
Offset and thermal drift of offset compensations for negative (**left**) and positive (**right**) post-manufacturing offset.

A technique used to reduce the so-obtained thermal drift is then fully described in the literature [16]. To summarize, two resistors, a parallel *R_p_* and a serial *R_s_* (Figure 5), are added to compensate for offset with a reduced impact on TDO. *R_s_* and *R_p_* are fabricated in same process and so, have the same TCR and, additionally, this coefficient must be as low as possible to reduce second order effects. *R_s_* and *R_p_* must be added in one of the bridge’s branches according to the initial sign of the offset as depicted in Figure 5.

For the so-obtained modified half bridge, thermal sensitivity of *V_out_* is minimum if [16]:

(2) $$R_{s}R_{p}=R_{1+}R_{2-}\approx R_{0}^{2}$$

The value of *R_s_* can be easily calculated to compensate half of the initial offset. The so-obtained value is given by:

(3) $$R_{s}=\frac{2V_{offset}R_{0}}{V_{dd}}$$

Then, *R_p_* is calculated from Equation (2) and compensates for the remaining half of the initial offset. 

This technique has two main limitations. First, the relationship between *R_s_* and *R_p_* is nonlinear. Therefore, if *R_s_* is a linear digital potentiometer, *R_p_* cannot be controlled linearly. Second, for a sensor with a small offset, the required value of *R_s_* to compensate half of the offset is very small (e.g., $R_{s}=R_{os})$, and so a huge value of *R_p_* may be required ($R_{p}=R_{0}^{2}/R_{os}$). Such a large resistance will be difficult to integrate in a CMOS circuit. 

**Figure 6:**
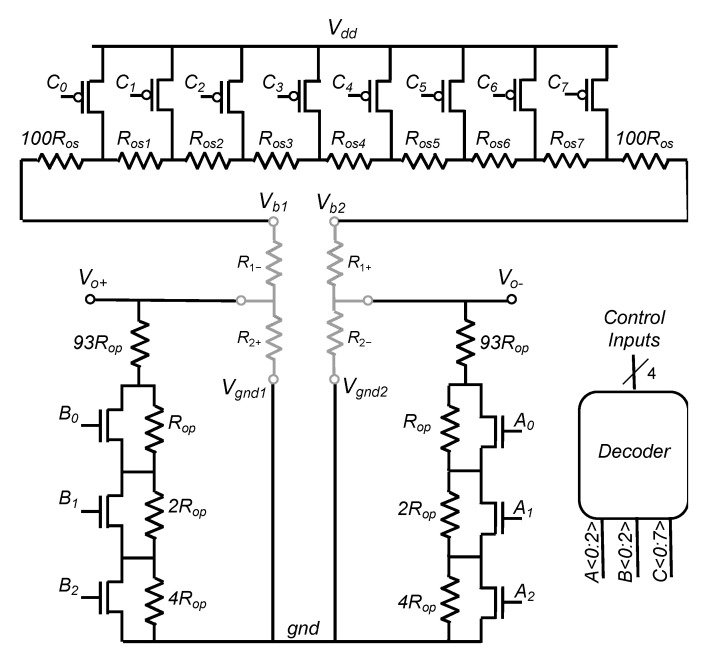
Improved architecture for cancellation of both offset and TDO with *n* = 7.

To overcome both previously mentioned limitations, we propose an efficient modification of the state-of-the-art solution [16] based on a theoretical analysis that concludes to the addition of a constant resistance in series with each branch of the WB. Those resistances must be small with respect to the nominal resistance of the WB, *R*_0_, but large compared to the elementary resistors, *R_os_*, used to compensate for the offset. The so-obtained modified architecture is illustrated in Figure 6, where a pair of resistances equal to 100*R_os_* are added to the digital potentiometer, between *V_b_*_2_ and *R_os_*_7_ and between *V_b_*_1_ and *R_os_*_1_. The remaining part of this section focusses on this adapted implementation and on the analysis of its performance based on Monte Carlo simulations.

Let us assume that a serial resistance is added in both WB’s branches which value is given by:

(4) $$R_{s}=100R_{os}+kR_{os}$$

where *k* is an integer ranging from 0 up to *n, n* being the number of elementary *R_os_* implemented in the digital potentiometer. Using both Equations (2) and (4), $R_{p}$ can then be approximated by the first term of the Taylor expansion:

(5) $$R_{p}=\frac{R_{0}^{2}}{100R_{os}+kR_{os}}=\frac{R_{0}^{2}}{100R_{os}\left(1+\frac{k}{100}\right)}\approx \frac{R_{0}^{2}}{100R_{os}}\left(1-\frac{k}{100}\right)$$

With $R_{op}=\frac{R_{0}^{2}}{10000R_{os}}$, the value of $R_{p}$ is then given by:

(6) $$R_{p}\approx 100R_{op}-kR_{op}$$

Therefore, the maximal value of *R_p_*, obtained for *k* = 0, is reduced by two orders of magnitude with respect to the original implementation [16]. This is obviously interesting for an IC implementation of this principle. Additionally, both parallel resistances can now be controlled linearly with discrete steps of one *R_op_* from 100*R_op_* down to (100 − *n*) *R_op_*. In addition, the control of the two additional digital potentiometers, namely *A* and *B* on Figure 6, does not require any additional configuration bits as *A_i_*, *B_i_* and *C_i_* are generated from the same control word. Indeed, for both branches of the WB, when *k* elementary *R_os_* are added serially, *kR_op_* have to be removed in parallel. In the example of Figure 6, *R_p_* resistors are implemented as two digital linear potentiometers in parallel with *R*_2−_ and *R*_2+_ and they can be linearly adjusted from 100*R_op_* down to 93*R_op_*. Finally, all three potentiometers *A*, *B* and *C* are controlled by a single digital word of four bits that allows them to encode 15 possible configurations.

The procedure of offset compensation is then modified as follows: initially, all switches of potentiometers A and B are opened, and switches of potentiometer C are closed. When *R_os_*_1_ is added (respectively, *R_os_*_7_) in series with *V_b_*_1_ (resp. *V_b_*_2_), one *R_op_* is removed from potentiometer B (resp. A); this procedure is repeated until the offset sign changes. 

**Figure 7:**
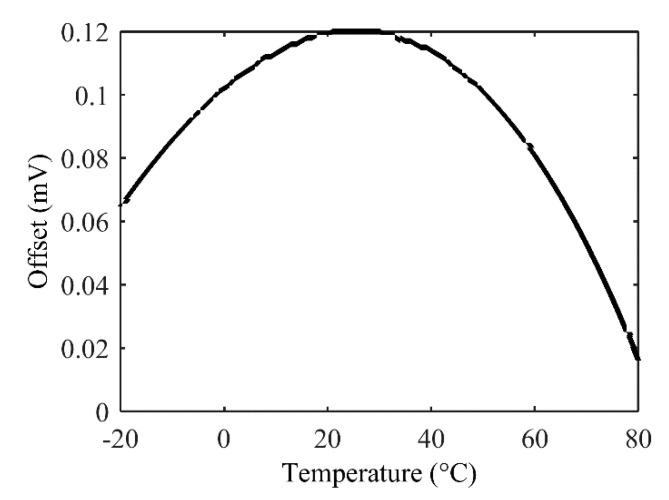
Typical TDO after offset compensation with the improved architecture.

Electrical simulations of the modified architecture have demonstrated the same level of both offset reduction and PSRR improvement as presented in Table 1. A major improvement of the thermal drift is illustrated in Figure 7: TDO is reduced down to about 100 μV over a −20 °C up to 80 °C temperature range. MC simulations demonstrate a maximal drift that has been divided by a ratio of about 6 and a log-normal distribution. It can be noticed that adding serial and parallel resistors to the sensing resistors may decrease the sensor sensitivity of about 10% in average. It is then assumed that the sensor has a higher initial sensitivity than required by the application. 

## 4. Automatic Fine Offset Compensation

In addition to the previously presented offset compensation circuitry, an automatic offset calibration procedure is added to compensate for residual offset and to ensure long sensor life by finely adjusting offset at each power-up of the circuit or upon request depending on the application. This compensation is based on a digital potentiometer similar to potentiometer *C* and connected between terminals *V_gnd_*_1_ and *V_gnd_*_2_. Depending on application, auto-zeroing process may start when power supply is ramping-up, if a zero-input is then guaranteed, or when a specific input is set, if an independent auto-zero procedure is required. The proposed procedure involves a digital finite-state machine (FSM) controlled by the sign of the sensor differential output that sweeps all combinations and stops when offset sign has changed.

Illustration of this additional digital potentiometer comes later in this paper. It is composed with *n* resistors, *R_F_*_1_…*R_Fn_*, and *n* + 1 control switches (*D*_0_…*D_n_*). Note that resistance of each elementary *R_Fi_* is small compared to *R_os_*, typically *R_os_* = *nR_Fi_*. Therefore, the impact of this new serial resistance on TDO is negligible. The automatic procedure uses an on-chip comparator to determine offset sign (positive or negative). Obviously, a comparator with a very low offset and a small thermal drift is required. Back to the on-going example of application, the remaining offset after coarse tuning lies in the range of ±1.55 mV (Table 1), a maximum final offset equal to ±220 µV should be obtained using seven *R_F_* resistors (controlled by four bits) and an ideal comparator. Note that the theoretical improvement of the offset is only *n* (vs. 2*n* for the coarse tuning presented in Section 3) due to the systematic over-compensation of the automatic procedure; indeed, the choice of the best code would require an ADC to determine the minimum of offset rather than a change of sign of this offset thus leading to a complexification of the FSM [6]. The next sub-section addresses the straightforward design of a comparator with low offset.

### 4.1. Design of a Simple Low-Offset Comparator

**Figure 8:**
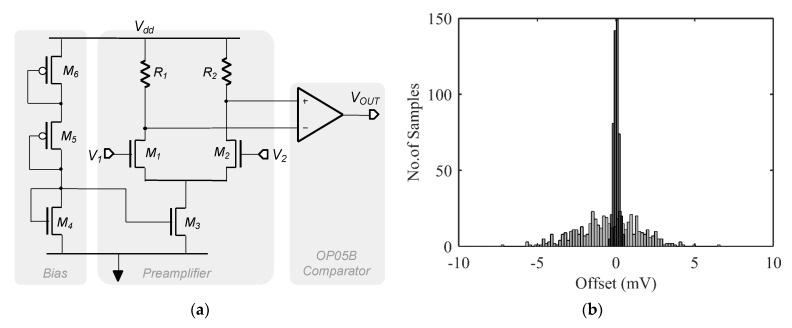
(**a**) A simple design to reduce offset of an existing comparator, (**b**) Monte Carlo simulations of input-referred offset of comparator IP (in grey) and a pre-amplified comparator (in black).

Using a standard Miller Operational Amplifier, from the analog IP library of the technology (AMS CMOS 0.35 μm), as a comparator, the automatic procedure leads to a degradation of the maximum final offset after fine tuning. This is due to the amplifier offset that is much larger than the targeted offset. From the datasheet of the IP, this phenomenon is obvious as the offset of the operational amplifier is guaranteed between ±7 mV. To fix this, an additional differential gain stage is added as a preamplifier to a standard Miller Operational Amplifier, namely OP05B, as depicted in Figure 8a. It is based on a NMOS differential pair *M*_1_ and *M*_2_ (*W* = 1 mm and *L* = 10 μm) and a pair of resistors, *R*_1_ and *R*_2_, equal to 615 kΩ. *M*_3_ sets the bias current of the amplifier by copying the current provided by the branch of *M*_4_*, M*_5_ and *M*_6_. Therefore, the input-referred offset of OP05B is divided by the preamplifier gain and this resulting offset is added to the input-referred offset of the preamplifier. To limit the preamplifier offset and 1/*f* noise, large dimensions have to be used to keep the symmetry of the differential pair and to avoid mismatches between both inputs. A smaller area alternative would be to consider well-known architectures [17] such as chopper stabilization, correlated double-sampling or auto-zero.

A set of 500 Monte Carlo simulations is then used to compare the OP05B IP with the so-obtained low-offset comparator in terms of input-referred offset. With a typical preamplifier gain of 25, an input-referred offset of ±300 μV is observed, thus resulting in a reduction of the offset by a factor of about 20. This performance level is sufficient for a proof of concept, even if further developments may concern the design of a specific low-offset comparator avoiding the use of a standard Miller operational amplifier and thus limiting silicon area and power consumption while improving residual offset.

### 4.2. Residual Offset after Fine Tuning

**Figure 9:**
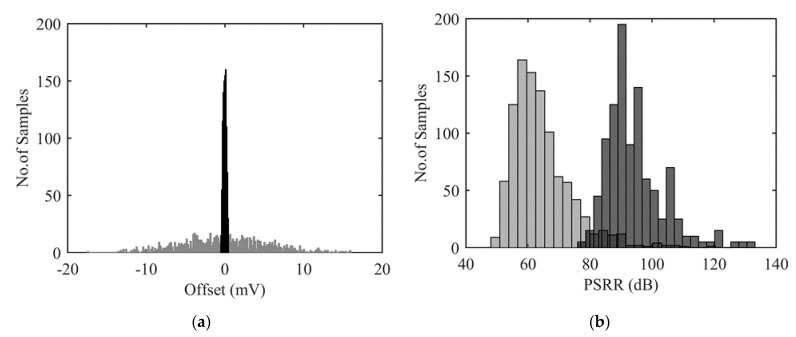
Monte Carlo simulations: Offset (**a**) and PSRR (**b**) are reported for a WB before (in grey) and after (in black) offset compensation.

Using the previously proposed comparator and an automatic procedure to implement a fine tuning of the residual offset, a maximum final offset of ±500 µV is obtained from a set of 1000 Monte Carlo simulations (cf. Figure 9a). This value corresponds to the comparator offset plus the fine-tuning discretization induced by switching one resistance *R_F_*. Additionally, the minimum PSRR is increased up to 78 dB, as illustrated in Figure 9b. With respect to the architecture without fine tuning of offset (Table 1), a significant improvement is observed: offset is divided by 3 and PSRR is increased by 8 dB.

Last but not least, design must pay attention to monotonicity of linear potentiometers. This is particularly true for the fine-tuning potentiometer that involves very small elementary resistances, *R_Fi_*, of about 1 Ω. This value must be greater than the maximum possible mismatch between the on-resistance of two consecutive switches, *D_i_* and *D_i+_*_1_. This mismatch has been characterized using MC simulations and it appears that a worst-case ∆*R_on_* of 164 mΩ has been obtained for transistor with $\frac{W}{L}\left(MOS\right)=\frac{50\;\mu m}{0.35\;\mu m}$. 

## 5. Scale Factor Adjustment

**Figure 10:**
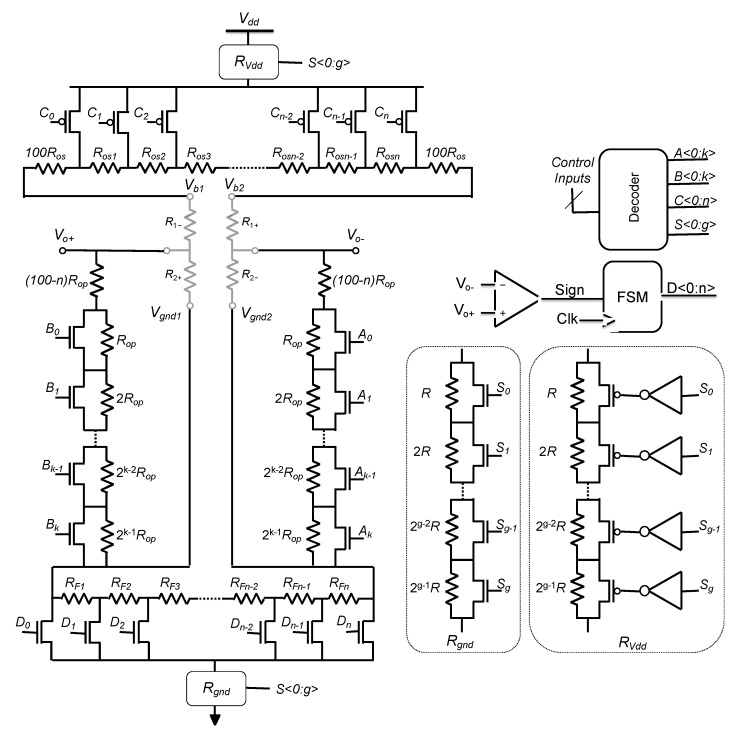
Complete architecture of the proposed IP for post-fabrication calibration of resistive sensors.

According to Equation (1), output voltage of a given sensor depends on supply voltage, *V_dd_*, bridge nominal resistance, *R*_0_, and resistance variations, Δ*R*. Since Δ*R/R*_0_, for a given input magnitude, is generally set by fabrication, and is independent of *R*_0_, WB output voltage may be adjusted linearly with supply voltage. In general, depending on application, sensors have a scale factor requirement. Then, if the initial value of the scale factor is higher than this requirement, downscaling may be applied using two potentiometers, *R_gnd_* and *R_Vdd_*, as depicted in Figure 10.

**Figure 11:**
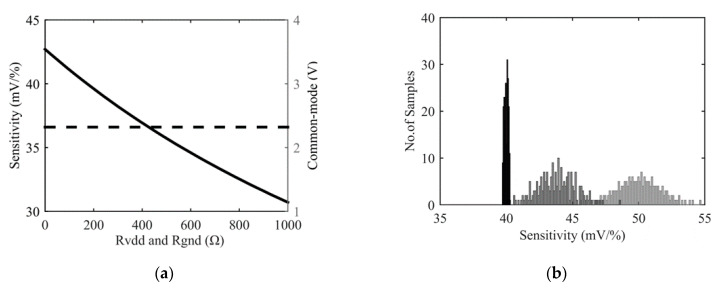
Results after scale factor adjustment: (**a**) variation of sensitivity (continuous line) and common mode voltage (dashed line) with *Rgnd* and *R_Vdd_*, (**b**) Monte Carlo simulation of sensitivity of a bare Wheatstone bridge (light grey), an offset compensated Wheatstone bridge (dark grey) and a fully compensated Wheatstone bridge (in black).

According to (1), a sensitivity of 50 mV/% is obtained for a full bridge configuration and a supply voltage of 5 V. After offset compensation (i.e., coarse and fine tuning), the mean value of this sensitivity is reduced up to about 10% (45 mV/%) due to the presence of compensation resistances that are added in series and parallel with sensor resistors. To mimic the impact of process scattering on sensitivity, a gaussian distribution with a 10% maximal deviation of the so-obtained scale factor has been modeled. Consequently a σ of 3.33% of the mean value is observed for the scale factor. To reduce this dispersion, any value of the scale factor, lower than the minimum of the MC distribution, can be targeted. As an example, a sensitivity target of 40 mV/% is chosen. To reach this target, both values of *R_gnd_* and *R_Vdd_* are increased symmetrically from 0 to 930 Ω using five configuration bits and elementary steps of 30 Ω. An example is presented in Figure 11a where the sensitivity target is reached for a resistance value of 180 Ω. It can be noted that common mode voltage is constant during this procedure. Normally, this value should be *V_dd_*/2 but it is slightly decreased during coarse tuning of offset due to the added resistors between *V_dd_* and *V_b_*_1_, *V_b_*_2_ and to digital potentiometers A and B that are added in parallel with *R*_2+_ and *R*_2−_ (Figure 10). It is worth noting that precise control of the common mode can be added at the price of two independent control signals for *R_gnd_* and *R_Vdd_*. 

Results of Monte Carlo simulations are presented in Figure 11b. They confirm the effectiveness of the procedure. The targeted scale factor of 40 mV/% is reached and relative variations of the sensitivity are reduced by a factor 10 (1% vs. 10% in the worst case). As for previously described calibration procedures, the residual uncertainty of the scale factor may be reduced by increasing the number of bits to control both *R_gnd_* and *R_Vdd_*. 

Table 2 summarizes significant results that have been reported in this paper. The first step consists in inserting a coarse tuning of offset block (CTO, potentiometer C) that allows a significant reduction of the offset and an associated improvement of the power supply rejection ratio (PSRR). The price to pay is then a drift of the offset with temperature that can be as high as 6.5 mV for a 100 °C variation of temperature. Drift of offset with temperature has been significantly reduced, by one order of magnitude, after adding a temperature drift reduction module (TDR, potentiometer A and B) controlled by the same configuration bits than the CTO block. An automatic module is then used for fine tuning of offset (FTO). An internal finite state machine is used at request to implement an auto-zeroing procedure that allows us to reduce the offset with a ratio of 30 compared to a bare WB. Additionally, the PSSR is increased up to a minimum of 78 dB, thus representing a 30 dB increase with respect to a bare WB. Last but not least, a module for the centering of the scale factor has been added to the architecture (SFA) to reduce scale factor scattering by one order of magnitude. This last addition also reduces the temperature drift of offset due to a reduction of the effective power supply voltage applied to the WB.

## 6. Experimental Results and Discussion

**Figure 12:**
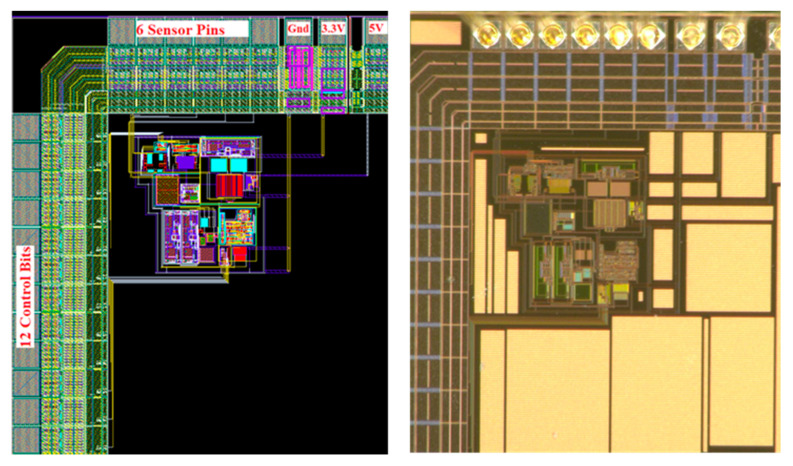
First demonstration IC for post-fabrication electrical trimming of resistive sensors: layout view (**left**) and photograph (**right**).

A first demonstrator has been fabricated in a 0.35 μm Technology from AMS (Figure 12). For digital potentiometers, we have used various polysilicon layers according to the requested elementary resistances and taking into account required temperature coefficients. The so-obtained design parameters (with respect to Figure 10) are:

- Offset compensation range: ±30 mV,
- Power supply voltage: 5 V,
- Nominal resistance in the Wheatstone bridge: *R*_0_ = 5 kΩ,
- For potentiometer C, design choice consisted in implementing a rpoly2 (50 Ω/sq.) resistance n*R_os_* = 75 Ω with *n* = 15 and a rpolyh (1 kΩ/sq.) resistance 100*R_os_* = 500 Ω.
- As a consequence, potentiometer A and B implements a resistance ranging from 41 kΩ up to 50 kΩ in n steps. To cancel-out effect of temperature coefficient of resistances, rpoly2 and rpolyh are also used accordingly to potentiometer C.

Moreover, a seven-bits linear potentiometer with a maximum resistance of about 7.5 kΩ has been added to adjust sensitivity, and an internal FSM with 32 states has been used to control a linear potentiometer with 14 resistances of 1Ω (rpoly1, 8 Ω/sq.).

ASIC is less than 500 × 500 µm^2^ in silicon area. Most of the silicon is occupied by control I/Os that have been implemented as a parallel bus in this first demonstrator. Obviously, a future version of the smart-trimming IC will include an SPI control and on-chip non-volatile memories to reduce both footprint and numbers of wires to be bounded. A rough evaluation allows to estimate the size of the die that could be used to replace the laser trimming die to about 1 mm^2^ including I/Os in this technology.

**Figure 13:**
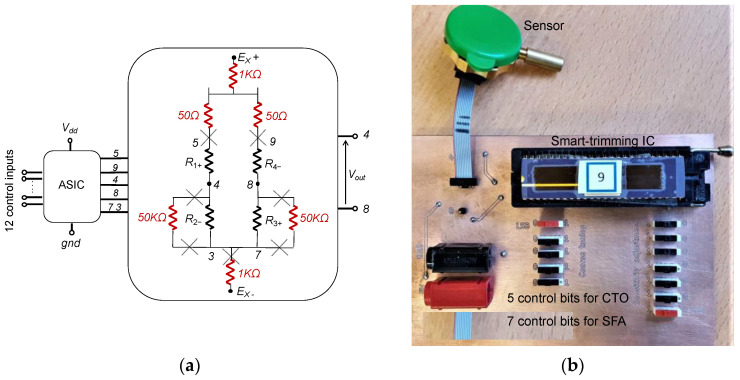
Proof of concept demonstrator with smart-trimming IC and a commercial piezo-resistive pressure sensor: (**a**) schematic, (**b**) printed-circuit board.

For the purpose of the proof of concept, we have used a commercial piezo-resistive pressure sensor that includes in a single package the sensor and a laser-trimming die. The so-obtained internal electrical schematic (Figure 13a) is composed of sensor’s resistors—namely *R*_1+_*, R*_4−_*, R*_2−_ and *R*_3+_—and the trimming network resistors depicted in red on the schematic with their initial values before laser-induced increase. After disconnecting the trimming network, we connected the proposed smart-trimming IC to the bare sensor, as illustrated on Figure 13a, to obtain a demonstrator board illustrated in Figure 13b. As pins 3 and 7 of the sensor are physically connected, the automatic fine-tuning procedure will not be applicable on this demonstrator.

**Figure 14:**
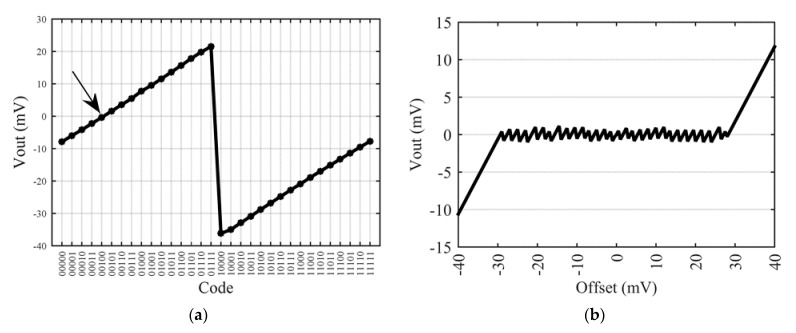
Measured output voltage of the smart-trimmed sensor after coarse tuning: (**a**) sensor output voltage vs. 5-bits code with arrow representing optimal code for this example, (**b**) sensor output voltage vs. initial offset.

After removal of the trimming die, the bare sensor offset has been measured at around −8 mV at ambient temperature. Figure 14a reports the evolution of this offset versus the applied CTO code. For code 00000, corresponding to a null impact of the CTO, the bare sensor offset is obtained. Adjustment steps of about 2 mV per code are also verified. In this example, the lower residual offset is reached for code 00100 with a value of −450 µV.

To verify the full range of operation of the CTO, we added a linear potentiometer in one branch of the Wheatstone bridge to emulate sensors with initial offset between −40 mV and −8 mV for code 00000. Then, putting the linear potentiometer in the other branch, initial offset between −8 mV and +40 mV are emulated. For each position of the potentiometer, the initial offset is measured with code 00000 and the residual offset after compensation with the best code is reported versus the initial offset (Figure 14b). Experimental results demonstrate the expected ability of the proposed trimming architecture to compensate offset in the specified range ±30 mV and to reduce it by a factor up to 2*n* = 30 down to ±1 mV after compensation. 

**Figure 15:**
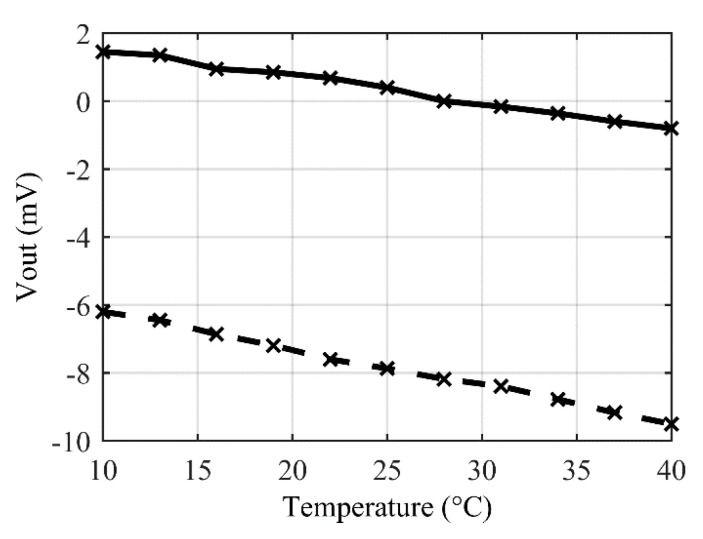
Temperature drift of offset: bare original sensor (dashed line) compared to smart-trimmed sensor (continuous line).

Then, TDO has been characterized (Figure 15). For the bare sensor a temperature drift of −106 μV/°C (dashed line) is measured. As a bare WB is not supposed to have a thermal drift of offset, this thermal drift is probably due to a thermal cross-sensitivity linked, for example, to a thermal deformation of the membrane. After connecting the sensor to the smart-trimming IC, experimental results (continuous line on Figure 15) demonstrate a small improvement of the temperature drift of offset (TDO) down to −75 µV/°C. This result confirms the theoretical expectations and proves that the proposed CTO does not degrade thermal drift. A small improvement of the TDO has even been observed due to a small reduction of the scale factor when inserting serial and parallel resistors in the bridge. It is worth noting that taking into account a previously characterized thermal-sensitivity during IC design would allow to cancel-out this cross-sensitivity.

**Figure 16:**
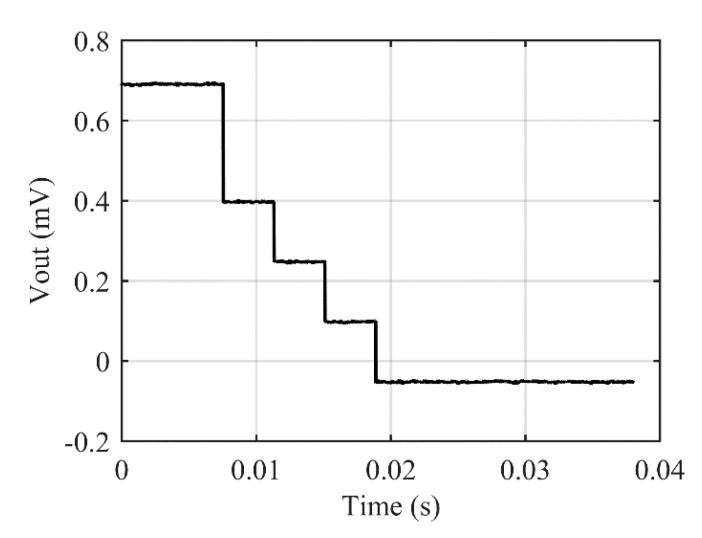
Auto zero procedure starts automatically at power-up to cancel-out most of the residual offset.

As previously mentioned, automatic fine-tuning of offset cannot be validated on this first demonstrator. A specific experiment based on four identical resistors of 4.7 kΩ is then used to verify the fine-tuning steps, the internal clock frequency and the comparator offset. Figure 16 illustrates a typical auto zero procedure at power-up. At *t* = 0, output voltage is equal to +700 µV due to mismatches between the four discrete resistors and the FSM starts decrementing output voltage by steps of about 150 μV till the output voltage sign changes. It can be noticed that the first step is twice the others due to a mismatch effect at the boundaries of the network of resistors. This effect could be reduced or even cancelled by a redesign of the layout and the addition of dummy structures at the boundaries of the network of resistors. The internal clock frequency is characterized from the width of each steps: the clock period is equal to about 4 ms corresponding to a clock frequency of about 260 Hz. On this specific example, the residual offset after fine tuning of offset is equal to −58 μV.

Finally, sensitivity adjustment has been verified. This electrical trimming is controlled by a seven bits digital signal to control potentiometer *R_gnd_* and *R_Vdd_* on Figure 10. Both resistors are adjusted between 0 and 7620 Ω in 128 linear steps of 60 Ω: the so-obtained adjustment range scales from 25% up to 100% of the initial sensitivity. This feature may be interesting for application requesting accurate and calibrated scale factors while typical fabrication technology implies scatterings with standard deviation in the range of 10%. Obviously, this results in a strong reduction of the standard deviation at the price of a lower average value.

Overall, experimental results obtained with this first demonstrator are in-line with our expectations. By implementing coarse and fine tuning of offset, offset can be reduced by a ratio of about 2*n*^2^ using only two digital potentiometers with *n* discrete elements. Integration, after analysis and improvement, of a state-of-the-art solution for cancelation of thermal drift of offset has been also validated thus allowing to significantly improve the minimum detectable signal at the output of a WB. Scale Factor calibration has been also implemented independently to further improve specifications of a COTS sensor. It is worth noting that even if the architecture is generic, an ASIC design may not be optimal for any sensor. For example, if resistances of several tens or even hundreds of kΩ are implemented in the sensor, integrated potentiometers must be adapted to the post-calibration requested performance in terms of offset, PSRR and scale factor. Last but not least, ageing compensation and re-calibration is possible for high-end sensors. To conclude, our proposal is particularly adapted for sensors that must behave as passive ones with performances of carefully calibrated ones.

## 7. Conclusions

In this paper, a fully electrical architecture for smart post-fabrication trimming of resistive sensors has been proposed. It allows to easily compensate the impact of process variations on a resistive sensor thanks to a compact IC. The proposed architecture is generic and may apply to any resistive sensors with six independent terminals (i.e., with independent *gnd* and *V_dd_* terminals for each branch). Once connected to the sensor die, potentially in a single package, the so-obtained smart-sensor still looks like a four-terminal Wheatstone bridge and it is then possible to adjust both output offset and sensitivity using digital control inputs accessible only during calibration. Additionally, an on-chip fine-tuning of offset may be automatically launched on power-up or upon external request depending on the application. Degraded modes are possible such as, for example, if the ground terminal is common to both branches, as in the presented experiment, the fine tuning of offset at each power-up or upon request is no longer available and, for a fully connected Wheatstone bridge with only four terminals, scale factor adjustment, using *R_Vdd_* and *R_gnd_*, and partial offset compensation, using potentiometers A and B, remain available.

Electrical simulations based on intensive Monte Carlo simulations have demonstrated the main advantages of the proposed solution: fully electrical operation, significant reduction of offset and PSRR, fine adjustment of sensitivity. Application to a COTS five-terminal sensor connected to an ASIC demonstrator, fabricated in a 0.35 μm Technology from AMS, has been demonstrated successfully, and thermal drift of the smart-sensor offset has been identified to remain identical to the bare sensor one.

Perspectives of this work concern different aspects to increase genericity and performance. First, the coarse tuning of offset can be adapted to cancel-out the offset thermal drift of the bare sensor if the theoretical work presented in Section 3 takes into account a model of this thermal drift. Linearity of the sensitivity adjustment may also be improved. Second, the number of configuration bits can be increased to reach higher levels of performance and to cover a large range of sensors with different values of nominal resistances. One can also imagine, in a future release of the IC, to include a serial-peripheral interface (SPI) to control post-fabrication trimming with an extended number of bits. This SPI will be used to control the internal logic state and, after calibration, anti-fuse [13] will be burned to freeze the IC configuration individually for each sensor. Non-volatile memories [14], including emerging technologies such as Magnetic RAM [15], could be alternatively used to provide reversible operation and on-field calibration.

## Figures and Tables

**Table 1:** PSSR and offset obtained from MC simulations after coarse tuning of offset with 7 elementary resistors.

Monte-Carlo Simulation Results				
	Min	Max	Mean	Std-dev
Offset (mV)	−1.55	1.35	0.080	0.766
PSSR (dB)	70.5	118	n.a.	n.a.

**Table 2:** Performance level of the proposed solution for post-manufacturing calibration of resistive sensors (MC simulations).

Performance/Inserted Modules	Bare WB	CTO (4 Bits)	TDR (4 Bits)	FTO (4 Bits)	SFA (5 Bits)
Offset (σ, mV)	5.65	0.766	0.766	0.179	0.179
PSSR (min, dB)	48	70	70	78	78
Temp. drift of offset (max, mV)	0	6.5	0.68	0.7	0.56
Scale Factor (V_out_ @ 1%, mV)	50.0	48.6	43.7	43.0	40.0
Scale Factor uncertainty (σ, %)	3.3	3.3	3.3	3.3	0.3
